# Following in Elders’ Footsteps: Yarning About Ageing Well in the Torres Strait

**DOI:** 10.1177/10497323251335210

**Published:** 2025-04-28

**Authors:** Rachel Quigley, Chenoa Wapau, Betty Sagigi, Sarah G. Russell, Sean Taylor, Sarah Larkins, Edward Strivens, Michelle Redman-MacLaren

**Affiliations:** 1 College of Medicine and Dentistry, 8001 James Cook University, Cairns, QLD, Australia; 2 2113 Cairns and Hinterland Hospital and Health Service, Cairns, QLD, Australia; 3 157852 Torres and Cape Hospital and Health Service, Thursday Island, QLD, Australia; 4 Melbourne School of Population and Global Health, 2281 University of Melbourne, Melbourne, VIC, Australia

**Keywords:** First Nations, Torres Strait, ageing well, cultural determinants of health, yarning

## Abstract

There is a growing body of literature around ageing well for Indigenous Peoples internationally. However, the perspectives of Torres Strait Islander Peoples, one of two First Nations groups in Australia, have not been documented. This qualitative study aims to explore what ageing well means to people living in the Torres Strait and Northern Peninsula Area (NPA) of Australia. Ten yarning circles were conducted with 45 participants from four island and five NPA communities across the region. Reflexive thematic analysis was used to identify seven themes of ageing well. A metaphor of a wongai tree—an endemic Torres Strait region tree—was used to describe those findings. The roots were used to represent the Torres Strait Islander way of life. The trunk represented practicing Torres Strait Islander identity. The branches represented a holistic approach to living a healthy lifestyle. The leaves represented strong leadership and role models. The fruit depicted passing on knowledge, tradition, and cultural practices. A cyclone, an adverse event, represented the challenges to ageing well, with the regrowth representing strong sustained life. Findings highlighted the importance of the cultural determinants of health, which significantly contribute to ageing well. These cultural determinants must be considered when addressing the health of First Nations Peoples, and as such, First Nations voices must be central in the design and implementation of practices and policies that affect them.

## Introduction

Around the world, people—including Indigenous Peoples—are living longer (WHO, 2018). By 2050, the World Health Organization (WHO) estimates that the number of people aged 80 years and over will be more than 426 million—triple the number in this age group in 2020 (WHO, 2018). However, increased longevity does not always equate to prolonged good health (WHO, 2015). How well we age can, in part, be attributed to the cumulative impact of health inequities across the lifespan. Inequities can be specifically linked to physical or social environments, but can also result from barriers that affect opportunities, decisions, and behaviors (Sadana et al., 2016; WHO, 2015). Sadana et al.’s (2016) work, highlighting the impact of inequities and how they shape the health trajectory into older age, informed the WHO World Report on Ageing and Health (WHO, 2015). Findings emphasized the social determinants of health (SDoH) affect ageing in several ways, including in the prenatal period and early childhood, where socioeconomic influences have direct or indirect latent impacts; through the cumulative health impact of social and economic disadvantage or discrimination over the life course; and intergenerational transmission of health inequities (Sadana et al., 2016).

Aboriginal and Torres Strait Islander Peoples are the two distinct First Nations Peoples of Australia. As with all ageing populations, Aboriginal and Torres Strait Islander Peoples seek to age well by remaining active, healthy, and independent for as long as possible (Department of Health, 2021). Health inequities are significant for First Nations Peoples, who have been negatively affected by the ongoing impacts of colonization and systemic discrimination for over two centuries. In addition to the SDoH, the Cultural Determinants of Health (CDoH) provide protective factors that mitigate this negative exposure for First Nations People (Department of Health, 2021; Finlay et al., 2020). The CDoH include First Nations Peoples’ ways of knowing, being, and doing that embody a holistic approach to health and well-being, enhancing resilience and strengthening identity (Department of Health, 2021; Finlay et al., 2020). They include connection to Country, family, kinship, and community; beliefs and knowledge; cultural expression and continuity; language; self-determination; and leadership (Bourke et al., 2018; Finlay et al., 2020).

In order to live long and healthy lives, First Nations Peoples must be at the center of designing appropriate health and social care services that support them to age well. The design of these services needs to incorporate First Nations Peoples’ world views, as the holders of cultural knowledge and practice, and must reflect the CDoH (Finlay et al., 2020), and thus merely adapting services that have been designed for and by other populations is inadequate (Finlay et al., 2020). Developing appropriate health and social care services that support ageing well can only be achieved if perceptions of, and priorities for, ageing well are voiced, acknowledged, and embedded into policy and programs (Department of Health, 2021; Quigley et al., 2022).

Globally, the perceptions of what Indigenous Peoples consider necessary to age well are mostly consistent, despite obvious cultural differences (Quigley et al., 2022). In a review of literature related to Indigenous Peoples worldwide, four major interrelated themes on ageing well were identified: achieving holistic health and well-being; maintaining connections; revealing resilience, humor, and a positive attitude; and facing the challenges (Quigley et al., 2022). Challenges included lack of access to housing, transport and adequate nutrition, and the impacts of colonization such as loss of language and intergenerational trauma (Quigley et al., 2022). The findings outlined how the concept of ageing well is enabled by spiritual, physical, and mental well-being, with reliance on connections to person, place, and culture. The literature also highlighted common challenges for Indigenous populations to achieve good health and well-being as they age. No literature in the review identified Torres Strait Islander Peoples’ perspective on ageing well, or identified the unique challenges faced by Torres Strait Islanders due to their unique cultural, social, religious, and geographic position (Quigley et al., 2022). In articles from Australia, participants were grouped as “Aboriginal and Torres Strait Islander.” However, there was no specific data attributed to Torres Strait Islander participants evidencing a need for further research in this area.

### Aim of Study

The aim of the study was to develop a framework to support ageing well for people living in the Torres Strait and Northern Peninsula Area (NPA). A participatory action research study was established to develop this framework. This paper reports findings from one qualitative component of the study that centralized the Indigenous research method of yarning and answered the following research question: “What does ageing well mean to First Nations Peoples living in the Torres Strait?”

This research is embedded within a larger body of work with the Healthy Ageing Research Team (HART). HART comprises Torres Strait Islander, Aboriginal, and non-Indigenous clinicians and researchers who have been delivering both clinical gerontology services and researching with Torres Strait Island and NPA communities for over 25 years. All studies have been developed through ongoing relationships and consultation with both health service staff, community, and local council groups over many years (Quigley et al., 2021). HART’s research is overseen by a specifically formed Knowledge Circle. This Knowledge Circle includes First Nations academics, community members, aged care workers, and health care staff who have expressed an interest in working with the research team on issues of ageing and health of older adults in their communities. Members share their expertise around study co-design and co-production, implementation, data collection and analysis, and dissemination of results; ensure research project methods and outcomes are culturally appropriate; take account of local issues; and ensure the involvement of local First Nations co-researchers to build research and health service delivery capacity in local communities. The group focuses on cultural content and ensures practices and values of their older people, their families, and communities are upheld during the research.

### Setting the Scene

The Torres Strait region lies between the northern tip of Queensland, Australia, and Papua New Guinea, and comprises over 100 islands with 18 communities on 17 inhabited Islands as well as two Torres Strait and three Aboriginal communities in the NPA of Cape York, on the mainland of Australia. The majority of the approximately 9000 people living in the region identify as Torres Strait Islander, a culturally, historically, and linguistically distinct group of people predominately of Melanesian ethnicity (Dudgeon et al., 2010). The islands of the Torres Strait are geographically divided into five main cluster groups: Top Western, Near Western, Central, Eastern, and Inner. Each cluster group has their own language or dialects and their own distinct history and cultural identity (Lawrence & Lawrence, 2004). Participants in the study were of Aboriginal and/or Torres Strait Islander descent living in communities in the Torres Strait and NPA, where most consider themselves to be living “a Torres Strait way of life” in reference to the region they live in rather than their identity. For this reason, we have respectfully used “Torres Strait” to reflect the geographical context of this article.

### Standpoint

The first author, RQ, is a non-Indigenous HART member who is leading the development of the framework for ageing well as part of her PhD. RQ is a clinician who has been working with Torres Strait communities for over 20 years, both in health service delivery and in research. The PhD study arose in response to feedback following HART’s previous research highlighting an increased prevalence of dementia in the region. Torres Strait community members recommended a move from a deficit approach to a more strengths-based approach to understand and develop a framework to support older people to age well. The authorship team includes HART members and the PhD student’s supervisory team and comprises Torres Strait Islander and non-Indigenous researchers with clinical backgrounds.

The larger research study, of which this qualitative component is a part of, takes a decolonizing approach and embeds Indigenous research principles into the methodology. Being inclusive of Indigenous ways of knowing, being, and doing is essential practice. Indigenous voices were brought to the forefront and a strengths-based approach, that promotes and celebrates the capacities and capabilities of the communities and individuals involved (rather than a problem-focused deficit ideology), was taken (Bryant et al., 2021; Fogarty et al., 2018). Torres Strait Islander team members were involved in all aspects of the research and brought Indigenous worldviews into the analysis of the yarning circles.

### Ethical Considerations

Ethical approval was obtained from the Far North Queensland Human Research Ethics Committee (HREC/2020/QCH/59342—1406) and James Cook University Human Research Ethics Committee (H8063). The study is aligned to the National Health and Medical Research Council guidelines on ethical conduct in Aboriginal and Torres Strait Islander health research (Commonwealth of Australia, 2018). All participants provided written consent prior to enrollment in the study.

## Methods

### Yarning as a Research Approach

Yarning is an Indigenous way of sharing knowledge through story telling (Barlo et al., 2020; Byrne et al., 2021; Murrup-Stewart et al., 2021; Walker et al., 2013). As a research tool, yarning facilitates in-depth discussions in a culturally safe place allowing Indigenous people to talk freely in an informal manner, providing rich data and in-depth descriptions on a particular issue (Bessarab & Ng’andu, 2010; Byrne et al., 2021; Smith et al., 2020; Walker et al., 2013). Yarning circles are recognized as an appropriate approach to research with First Nations People in Australia (Barlo et al., 2020; Bessarab & Ng’andu, 2010; Geia et al., 2013) and can be used to explore locally relevant knowledge that may better guide culturally responsive perceptions of health experiences (Geia et al., 2013; Walker et al., 2013). Data co-generated through yarning demonstrates rigor and a legitimacy of the research process for Indigenous people, as well as within the wider research community (Bessarab & Ng’andu, 2010; Shay, 2019). Using yarning circles as a research method centers Indigenous Knowledge systems, acknowledges the importance of relationality, as well as observing cultural protocols, and therefore facilitates culturally safe research (Atkinson et al., 2021; Barlo et al., 2020; Byrne et al., 2021; Shay, 2019).

### Yarning Circle Sites

Yarning circles were held at six sites from across the Torres Strait and NPA: Ngurupai Island and Kirriri Island (Inner cluster), Wug Community on Moa Island (Near Western cluster), Warraber Island (Central cluster), and Bamaga and New Mapoon (NPA).

### Yarning Circle Participants

Inclusion criteria: First Nations adults (aged 18 years and over) from four island communities and five NPA communities were included.

Exclusion criteria: People under the age of 18 were excluded. No other exclusion criteria were applied.

### Recruitment

Invitations to participate in the yarning circles were facilitated through health center staff, aged care services, promotion of the study on a local radio station, and recruitment flyers placed on community notice boards in local council offices, health centers, and community stores.

### The Yarn

Ten yarning circles were conducted, with a total of 45 participants. The yarning circles were co-facilitated by one or more Torres Strait Islander research team members (CW, BS, ST) and one or two non-Indigenous team members (RQ, SGR, ES). The composition of yarning circles was determined by participants and was specific to the circumstances of each community. In some communities, gender specific yarning circles were requested, so separate yarning circles were held for males and females, facilitated by male and female Torres Strait Islander researchers, respectively. In other communities, participants requested separate yarning circles for younger participants (in their twenties and thirties), and older participants rather than by gender. In some smaller communities, all participants, regardless of age or gender, wanted to yarn together.

Yarning circles commenced with a social yarn that included introductions to clarify relationships between and among participants and provided opportunities to develop trust and rapport with the researchers (Bessarab & Ng’andu, 2010). Refreshments were provided as part of the social yarn. Participants could choose if they would join in the subsequent research yarn, decide how long they stayed, and could end their participation at any time during the yarn—consistent with principles of self-determination.

The research yarns were opened with a Torres Strait Islander researcher asking participants, “What does ageing well mean to you?”. In some yarns, further prompts were required to focus the yarn on the barriers and enablers specific to the culture and traditional lifestyle that support healthy ageing, such as “What role does your culture play in being able to age well?”, and prevention of chronic disease and comorbidities, “How does your health affect you growing old?”. The role of environmental, cultural, spiritual, and other priorities for living well while ageing was also explored, with prompts that included: “How does living in this community support you to age well?”, “How does your family, friends, and community support you to age well?”, and “What are the things that are important to you as you grow older?”. The research yarns were audio-recorded with permission.

### Yarning Analysis

The data analysis method was guided by Braun and Clarke’s Reflexive Thematic Analysis (RTA) methods (2022). RTA is an interpretive approach to analyzing data across a range of theoretical frameworks, that facilitates the identification of themes across a given data set (Braun & Clarke, 2022). It addresses research questions that explore people’s experiences, perceptions, behaviors, and factors that influence a particular phenomenon as well constructing meaning of experiences (Braun & Clarke, 2022). In RTA, the researcher’s reflexive engagement with theory, data, and interpretation and the importance of the researcher’s subjectivity as an analytic resource are emphasized (Braun & Clarke, 2022). RTA is an appropriate method for analyzing yarning research (Murrup-Stewart et al., 2021). Braun and Clarke’s (2022) six phases that outline the process of RTA were used to guide the analysis for this research, incorporating additional processes for Indigenous involvement, as follows:

1. Dataset familiarization:

Verbatim transcripts were transcribed by RQ, de-identified and uploaded into NVivo 12 (QSR International) for data management. CW and BS (Torres Strait Islander researchers) translated any Torres Strait Creole that was spoken in the yarning circles, into English. During and after transcription and translation, the data were read and re-read by RQ, CW, and BS. Notes were made of any analytical observations.

2. Coding:

A collaborative coding approach was used to ensure Indigenous worldviews were incorporated into the understanding and interpretation of the data. A coding framework was developed by RQ, CW, BS, SR, and MRM based on the research questions, the initial observations of the data, and the literature. Transcripts were deductively coded by RQ using the framework. This initial coding of the data was undertaken systematically using a complete coding approach, where any item of data that might be useful in addressing the research question was coded. CW and BS reviewed and confirmed the coded data.

3. Initial theme generation:

In this phase, the coded data was explored to develop potential themes. The focus shifted to the interpretation of meaning across the whole data set. A mind-map was used to visualize the connections and relationships between codes with involvement from RQ, CW, BS, SR, and MRM. This generated the initial themes.

4. Developing and reviewing themes: Yarning about the yarning:

The initial themes generated became a yarning topic for the team to use to develop the themes more robustly. Using orality for data analysis upholds Indigenous research principles and paradigms (Mafile’o et al., 2024). The two Torres Strait Islander team members shaped the thought processes, generating new themes and combining others, intertwining Indigenous ways of knowing into the themes and providing Torres Strait Islander perspectives. Themes were discussed between RQ, CW, BS, SR, and MRM until consensus was achieved and the final themes derived. In this “yarning about yarning,” how well the themes provided an interpretation of the data that addressed the research question was also assessed.

5. Refining, defining, and naming of themes:

Continuing with the “yarning about yarning,” it was discussed how the themes should be presented through a Torres Strait Islander lens. As an oral centric culture, Torres Strait Islander people often organize and transmit knowledge around visual metaphors. These metaphors are concrete and explicit (physical, often nature-based objects) and are a common Torres Strait Islander way of explaining more abstract concepts in a comprehensible and relevant way (Mam et al., 1993). Metaphors are often grounded in land and story. As such, the idea was developed by the Torres Strait Islander researchers to present the findings of the yarning circles using the metaphor of a wongai tree. The wongai tree and its fruit is significant in Torres Strait Islander culture as a traditional food and carving material, and the seeds are used as jewelry. The wongai tree also features in Torres Strait Islander stories and there is a well-known legend which states that whoever eats the fruit of the wongai tree is destined to return to the Straits.

6. Writing up:

To align with the values of this study and our decolonizing approach, it was important that the write-up ended on a strengths-based narrative. Therefore, reporting the final theme of “Demonstrating strong sustained life: regrowth” focused on a positivity to ageing well rather than problematizing Torres Strait Islander peoples. The findings were also published in a plain language version, with wongai tree illustrations, as well as a visual animation video, for dissemination in communities across the Torres Strait and NPA.

## Findings

Ageing well, as expressed by participants, is represented by the life and structure of a wongai tree. Each theme generated relates to a part of the tree that best describes the findings. The themes are represented as (i) Living a Torres Strait Islander way of life: the roots, (ii) Practicing Torres Strait Islander identity: the trunk, (iii) Living a healthy lifestyle: the branches, (iv) Displaying strong leadership and role models: the leaves, (v) Passing on knowledge, tradition, and cultural practices: the fruit, (vi) Experiencing adversity: damaging events, and (vii) Demonstrating strong sustained life: regrowth. Representative quotes from the 10 yarning circles are included to center the voices of the participants and illustrate the findings.

### Living a Torres Strait Islander Way of Life: The Roots

The Torres Strait Islander way of life laid the roots for a healthy life and in turn healthy ageing. Participants described how connections to their island home, family, friends, and community, and interactions and support arising from those relationships, kept them strong and therefore supported ageing well. Those networks spread out extensively like the roots of the wongai tree, and like the wongai tree roots, supported all that grew above them.

A deep connection to participants’ roots—their island home—was explicit from all the participating communities. Being on traditional lands contributed to health and well-being: “Having a beach day, the beautiful view, the land, and the sea means so much to our health up here” (YC5). Participants wanted to grow old in their community and stay in their homes and on their traditional land: “I would rather stay here and get older” (YC9).

Connections to family as part of the Torres Strait Islander way of life were central and promoted ageing well. Being with family was a source of joy that kept people strong and happy: “Ageing well, it’s very simple, in my life experience, it’s [being with] my family” (YC10). Grandchildren in particular afforded older people with motivation to keep going and provided them with a purpose in life often through the responsibilities and structure needed to raise them. Grandchildren kept the older person active and on their feet.

[I] like to look at my grandkids and great grandkids. I’m happy with them. Looking at them as they are growing up and I’m growing old, they still make me happy. I get some energy from them; I am feeling good about them. (YC9)

Connections to the wider community as part of the Torres Strait Islander way of life were also discussed as promoting ageing well. Connections to, and contributing to, the wider community kept participants grounded, gave them a sense of purpose, were a source of joy, and provided participants with feelings of belonging:

They [older adults] actively engage, they actively laugh, they actively socialize, and that’s how I want to be when I get to their age, still a part of the community, still pulling my weight and making sure that community has a function. (YC1)

Community was also a great source of support both practically, “We still come together as a community and help each other out when need be and share things together” (YC3), and emotionally. One participant shared, “We sit and talk […] If I feel down, I talk to my cousin. And other people come to me, and we can talk about it” (YC8).

### Practicing Torres Strait Islander Identity: The Trunk

Torres Strait Islander identity, practiced through cultural activities and traditions, provided strength and well-being through the continuum of life and consequently supported ageing well. This theme is symbolized by the trunk of the tree rising from the ground. As the wongai tree grows, it has a direction and it takes on its shape, just as the identity of being a Torres Strait Islander is shaped through practicing tradition and culture, enabling growth, strength, and resilience. The trunk of the tree must be strong to combat the harsh sea winds, and be resilient against disease to have longevity. Likewise, traditions and culture need to remain strong for participants to age well.

Participants expressed how participation in cultural events such as island dancing, feasting, and craft activities facilitated growth, often through the opportunities to gain traditional knowledge, which contributed to ageing well: “Every time there was a feasting in our—like a gathering or a cultural activity,—I’m always there, learning knowledge” (YC1).

Participants saw the traditional lifestyle of “days gone by” as one of strength and good health. Participants reminisced about how the traditional ways of living promoted good ageing: “In the olden days there were no diabetics, no high blood pressure because of the way of living, the way they ate and everything, walking” (YC9). Participants talked about how it was a much simpler life, but often a harder life, for the older generations:

Life is very easy today and at your fingertips. Before people grew up with a very hard life, they had to get our food and the fuel to cook it. Now it’s just walk in a switch on the switch to get the light on, the food already [prepared] to eat. (YC9)

Participants described the physicality of everyday life in the past. People would do hard physical work in their everyday tasks such as collecting firewood for lighting and cooking, managing their gardens, carrying water from wells, hunting and gathering food, and rowing boats when fishing or for transportation. These activities kept them fit and healthy and contributed to their longevity:

I didn’t buy her coconut cans from the shop. I scraped, I cleaned and scraped coconuts. […] It’s only that little bit hard work but guess what you benefit from it? I’ve got muscles I never knew existed from scraping. (YC1)

Eating traditional food and traditional ways of sourcing food were seen as significant factors that contributed to ageing well, and were described as important in the past, with participants describing how their ancestors lived longer lives:

I remember as a kid my parents we would always live off garden food back in the days. I see my grandfather living with us and he had a good age. We never had dementia back then. Now we have the cancer and chronic conditions, more people are dying in the past few years from cancer. (YC10)

Most people would garden and eat food that they grew. That type of food was considered healthier, more satisfying, and less expensive than processed store-bought food used now: “The food that we have been grown up with would keep us healthy. People used to have gardens of their own with banana, cassava, sweet potato, pumpkin, and watermelon” (YC9). Ageing well was described as following the ways of your predecessors and returning to a more traditional way of living: “If we want to age healthily, we’ve got to follow our footsteps from before us especially if we want to stay strong and not fall by the way” (YC1).

### Living a Healthy Lifestyle: The Branches

A healthy lifestyle, which included physical, mental, cultural, and spiritual domains, was critical to ageing well. All aspects were connected, and if any one of those was lacking then it affected the health of a person. To age well required a balance across the domains and a holistic approach. A healthy lifestyle for participants is symbolized by the branches of the wongai tree. Branches grow in all directions and are different sizes, but they must be balanced—they don’t all grow from one side of the tree. If branches are missing, the tree becomes unstable and more susceptible to adverse forces:

[ageing well] It’s holistic. It’s the whole thing—culture, whether it’s spiritual, healthy eating, also wellbeing, individual wellbeing, psychological, but also socially as well, social with people. Interactions. Having everything, they all intertwine and makes a person. If one is out of balance the rest are unbalanced. For a healthy person I think everything needs to be all equal and level. (YC10)

Although living a healthy lifestyle was seen as a holistic concept, participants singled out specific elements of importance: mental health, physical activity, and diet, that influenced ageing. These are represented as balanced branches of the wongai tree.

#### Mental Health as a Branch

Participants described how having strong mental health was important to their overall health and supported longevity and quality of life: “I think the mental side of it is really powerful, it drives a person. So, to be healthy is to look after your mental wellbeing” (Y10). Participants emphasized the importance of discussing the significance of mental health with friends and family, as well as being open about any issues being faced:

If we have a problem we share. If we have a hard time with our husband, boyfriend, then we share. You can work out how you going to change this […] Going through domestic violence for me, I went really down, it was hard but thank God for my sista [sic] there. We would talk together. Mentally for me was, I was depressed. (YC8)

#### Physical Activity as a Branch

Another lifestyle factor that was singled out as a significant influence on ageing was physical activity. Some communities discussed how active their communities were, with organized sports such as darts, Australian football, rugby, Zumba, and island dancing, which encouraged community members to exercise: “When the football girls do their exercise, the community joins in too” (YC5). Others appreciated how the natural environment of their island communities was an ideal place for exercise, “The gym is the hill, the beach, the reef, the creek” (YC5), rather than formal venues or planned activities. The older generations were seen as good examples of remaining active and how that helped with ageing:

There are those elders who were up at dawn chucking a line off the reef here to catch fish for their children. These elders, they’re going to be around for a lot longer. Why—because they are still physically active. (YC1)

#### Diet as a Branch

Diet was discussed extensively across all communities as a lifestyle factor that influenced health and consequently impacted on how one aged: “The way we eat affects the ageing” (YC8). Participants discussed how a diet rich in fruit and vegetables and fresh fish, as well as portion-control, was considered healthier and contributed ultimately to longevity. It was emphasized that this information needed to be reinforced in the younger generation: “They [younger generation] need to know how to cook and grow their food […] we need to teach them about growing food traditionally and trying to get them off all the fast food” (YC2).

### Displaying Strong Leadership and Role Models: The Leaves

Strong leadership and role models within the community facilitated ageing well. This is symbolized by the leaves of the wongai tree. The function of leaves is to produce nourishment for the tree. Likewise, strong leadership provided sustenance to the community and played an important role in setting a moral compass and providing structure:

We have to lead by example. I’m approaching my Eldership now, we have to lead by example, and we have to lead in such a way that if they [the younger generation] see us healthy then they will be healthy. If they see us make the change, they will make the change. (YC1)

The importance of respect and moral values were also associated with leadership. The older participants appreciated and valued respect being shown to them, and felt it contributed to their overall well-being when ageing: “[being an older person] all of them nephews they listen to me when I ask them to do some things for me, so [ageing well] can be done if we have respect” (YC3).

### Passing on Knowledge, Tradition, and Cultural Practices: The Fruit

Passing on knowledge, tradition, and cultural practices was key to ageing well. This is symbolized by the wongai fruit. The main function of a wongai fruit is to spread the seeds (the nuts) contained in the fruit, to ensure continuation of its species. Likewise, the passing on (the spreading) of knowledge and culture is fundamental to the continuation of the Torres Strait Islander way of life and as such influenced the ageing trajectory. Passing on knowledge brought benefits not only from those that were learning, but also from those that were teaching. For the older adults, it gave them pleasure to know they were sharing their wisdom and skills: “It is important to me to pass on my knowledge and culture. Today all the boys say, ‘we should go and sit down with Grandad and learn. He will explain to us how to make the harpoon.’” (YC3). Passing on knowledge also provided older generations with a sense of purpose and fulfillment from being able to pass on skills and language to the young children and seeing their joy in learning from an Elder:

Them kids, they say, “nice to see you aka [grandma/name],” because they were happy to see me because of what I tell them, and explain for them, like, what’s true and what’s not true, and I [taught] them dances and song, and today I talk to my grandchildren, teach them lingo, and tell them what is right and what is wrong. (YC4)

The process of teaching kept the older person active and connected to their community: “We try to share this [traditional ways] in our women’s group, this year we are just started doing our women’s group. And to share those kinds of ideas to the younger ones” (YC8).

### Living With Adversity

Damaging events have resulted in adversity, impacting on Torres Strait Islanders’ way of life and identity, ability to live a healthy lifestyle, pass on knowledge, and maintain leadership roles. This theme describes how the impacts of colonization, religion, inequitable access to services, modern-day challenges such as the influence of social media and technology, and the broader SDoH have affected the ability to age well. This is symbolized by a damaging event to the wongai tree, such as a cyclone, that breaks off branches, blows off leaves and fruit, causes root damage, and exposes the internal trunk, allowing disease and rot to take place.

#### Impacts of Colonization as a Damaging Event

Participants likened colonization to a rot that had penetrated their society just like the rot of a tree. Ill health was described as a consequence of colonization: “We have a cultural hierarchal structure and practices which worked. Being tampered with have dismantled us slowly and surely and that then contributes to many factors that leads to ill health” (YC10). Participants particularly emphasized how the effects of historical trauma were impacting on the health of today’s generation, and how intergenerational trauma was influencing lifestyle decisions that affected health outcomes:

We are living, us as the third generation, we are living through what happened to the first generation before us. We’re just getting the tail end now hence diabetes and everything is coming through […] not only physical sickness but the mental sickness. The mental depression, those things are hindering our choices. They are the things; they are the actual barriers that stop us from making clear choices because you’ve got the trauma sitting in there. (YC1)

The impacts of colonization had a wider significance for participants than just on their health. Social breakdown had led to the loss of the traditional hierarchical structure within families and communities and with that, a loss of the teaching of cultural practices:

Torres Strait [Islanders] are cultural, traditional people, it’s only that we are going away from our traditional cultural lifestyle that we have ended up in this predicament, but when we were in that system of governance that we had in the community, the community was well, everybody was active, contributing. [Name] said to me, “Bala,[brother] one thing I notice, the old people back in the days, they had little, but they achieved much. Today we have much, and we achieve little.” (YC5)

#### Inequitable Access to Services as a Damaging Event

Participants from all communities described how access to aged care and health care services, and social, community, and recreational programs was, at times, problematic making ageing well a challenge. For participants, being able to access appropriate aged care services and therefore being able to grow old on their island community was very important to them, and for most residents the desire to die on island was significant. Remaining on their island home as they aged allowed them to remain connected to family, friends, community, and their land: “We want an aged care facility here on [community name] for our people, those that are getting ill and older, so they are not getting sent away [to a facility away from the community]” (YC10).

In only two of the communities did participants have access to a day respite center, but those able to access this service spoke of the benefits: “Coming here [day respite], getting your brain active, nice to share stories with all of my friends, having laughs” (YC9). For those in the more remote communities, the necessity to travel off community to access health care often resulted in participants either not accessing care or getting sub-optimal treatment: “Most of the people don’t want to go to their appointment, they scared of planes, and the weather is changing, raining all the time, they can’t go. Even go for screening, for breast screening, some go, some stay” (YC8).

#### Modern-Day Challenges as a Damaging Event

The cyclone also damages roots, which represents the weakening of the connections and support between families and communities. Participants described how the structure of modern-day society meant family members often had different priorities and responsibilities. For some, this meant having to leave the community for work and education, and consequently not being available to provide support to the older person, as this older participant stated: “At home I am by myself as the grandchildren are away working” (YC9). This breakdown in traditional family structure had implications for ageing well with regard to social isolation. For older participants, the lack of family presence left them feeling under-stimulated: “I am left at home by myself. Just sitting in my bedroom looking at four walls” (YC9).

Participants acknowledged that changes within a modern-day society came with challenges. The introduction of technology, including phones and TVs, and social media was seen as negatively influencing the traditional practices within the home and impacting on traditional lifestyles:

When we didn’t have a television, everybody would be out on the reef or the young boys would be making spears, the Elders would be showing the young boys how to make spears, but today when we have television and a lot of social media, the dynamics of the home have changed now. (YC5)

More generally, the introduction of technology was seen to be a deterrent for people being physical active. Participants observed that more people were staying at home and not interacting within the community and were less inclined to be active when they had access to technology: “It [technology] makes you sit at one place on the phone instead of exercising and doing stuff around the house …. technology slows you down, makes you not exercise” (YC7).

However, there was a realization from some of the participants that modern-day technologies were part of everyday life and a way to incorporate them into today’s culture was needed:

How do you grab what was practiced and what you aim to continue to practice like our culture, and incorporate the modern changing environment because, yes, we have to keep up with what is changing. How do you incorporate that and find a balance? (YC10)

#### Social Determinants of Health as a Damaging Event

Other aspects of life that were seen as a challenge of survival can be understood as the SDoH. Participants described the challenge of survival due to cost of living, lack of transport, housing issues, and environmental factors. The “high cost of living” (YC10) included costs associated with food, recreation, and transport. Transport between communities was problematic for some of the older participants that had to access the main hub of Thursday Island for health and aged care services:

I can’t go on the ferry, if I lost my balance […] I just can’t walk on […] and if I need to go to TI, [Thursday Island] my son has to take the day off from work and go over in my car on [the car ferry] and that costs money. And that’s why I only go once in a while. (YC4)

Ageing well encompassed more than health for the participants. They described how issues relating to housing affected their ability to age well. This included issues around overcrowding within homes: “The housing is overcrowded as well. For me and my family we have six in a 2-bedroom house, and it is not good […]. The only way is to relocate somewhere. But I don’t want to leave the island” (YC7). Environmental factors also impacted on some participants’ decisions to address lifestyle behaviors that could influence ageing. Many of the communities described the barriers to growing garden food. Some of the reasons included crops being eaten by wild horses, mice, and bush turkeys: “We can’t plant the veggies. Mainly only the cassava and sweet potato because the bush turkey dig it up” (YC9).

### Demonstrating Strong Sustained Life: Regrowth

Damage may have occurred following the devastating winds; however, the roots of the tree have not died, and there is still life in the tree and hope for the future as new growth sprouts forth. This is symbolic of the sustained strong existence of the Torres Strait identity and way of life attributed by the participants to resilience, positive attitudes, personal motivation, and taking responsibility for one’s health. This was also facilitated by activities to strengthen self-care, such as keeping occupied, doing the things that made them happy, and practising their faith.

Participants reflected on the Torres Strait Islander Peoples being historically resilient:

We should just stand up and say, OK, that’s enough, as a race, as a people. Because that’s not our style. We’re not that sort of people, we’re a resilient people, we stand up and we do things for ourselves. Maybe we need to go back there. (YC1)

Participants described having to overcome past adversity and take a positive approach to moving forward. For many of the participants, this meant taking responsibility for their own decisions and choices that affected their health. A change in lifestyle was required for a long and healthy life: “We have to be sensible and think about what sort of things that we put into our bodies” (YC4).

Personal motivation encapsulated the drive to stay active, fit, and healthy and in doing so remain independent:

She [older resident] keeps active and keeps herself going and I can see a couple of other [older] ladies that do the same thing. They’re keeping themselves […] even though they might be restricted in lots of things, but in other ways they’re keeping themselves going, motivated, motivated. (YC4)

Overall, participants took a positive attitude to ageing well and staying active and independent. For many, a positive attitude was expressed as looking forward to growing old: “I am looking forward to getting older. I’m always happy” (YC9). Passing on this attitude and setting a good example to the next generation was also important. The benefit of having this positive attitude was to be able to continue to stay well for their families: “I take a pleasure in trying to do the right thing. I try my best. That’s all I can do, for my kids, just try my best” (YC1).

Participants also described how doing activities that made them happy and provided inner strength ultimately supported ageing well. Activities that improved their well-being, avoided feelings of social isolation, and maintained self-esteem included interacting with nature, “I think ageing well for me is being at the seaside and doing my own things. Doing the things that I used to do with my parents, go bush, looking for bush food, and all them things” (YC10), and staying connected to friends and family, “[Being] with your grandkids, nephews and nieces, family members, best friends, [makes me happy]” (YC9).

Faith was seen as a way of staying strong and providing a sense of purpose in life, which contributed to ageing well. Faith provided pleasure and brought joy into people’s daily lives: “I go to church all the time. Every Sunday I go to church. All the other Christian people are there, and we sing, it’s nice” (YC9). Practicing faith also provided a shared interest to connect with friends and work colleagues and provided the opportunity to set good examples and pass on values and knowledge to the younger generations.

Generally, participants reported that keeping occupied was important for healthy ageing, whether this was through staying connected with friends and community members, participating in community events, partaking in cultural practices, or being active doing household chores: “If you sit you get lazy and you’re going down” (YC9). Participants discussed how keeping occupied had a physical focus, either through dedicated exercise or just through incidental exercise as part of everyday household activities: “I keep strong … working in the house, doing dishes and help my daughter” (YC9). Keeping occupied was also seen to assist with maintaining independence for the older participants: “I’m 79, I will be 80 next year, so I still do my things, do my washing, my cleaning and things like that, I never rely on my daughters, or my neighbours I do my things myself” (YC2). Keeping occupied was associated with personal motivation and attitude toward ageing: “I am 65 years old. But I still want to work because I want to stay fit and healthy” (YC3).

## Discussion

This study aimed to explore what ageing well means for First Nations Peoples living in the Torres Strait and NPA. Findings demonstrate that ageing well is more complex that just achieving good physical health or “Healthy” ageing. For participants in this study, ageing well encompassed a broader, more holistic view that included concepts absent from Western paradigms of healthy ageing models, but instead reflected CDoH. For First Nations Peoples, culture is the basis for health and well-being (Bourke et al., 2018; Finlay et al., 2020) and the strengths of culture have continued to evolve and thrive despite the negative influences (Department of Health, 2021).

The Torres Strait Islander way of life—through connections and relationships to family, friends, community, and island home—was at the heart of the yarns. This reflects the cultural domains of “Connection to Country” and “Family, kinship, and community” as described by Bourke et al. (2018) and Finlay et al. (2020) in their definitions of the CDoH. Indigenous Peoples’ connections to their traditional lands provide empowerment (Finlay et al., 2020) and are central to existence (Kingsley et al., 2013). For participants, the ability to age well was embedded in their connections to their island home or community (NPA) and disconnection from traditional lands compromised health and well-being. This finding aligns with global studies that describe how connections to Country for Indigenous Peoples influence the ageing trajectory (Browne & Braun, 2017; Butcher & Breheny, 2016; Pace, 2020; Radford et al., 2019). Connections to family friends and the community also had significance for the participants. Strong ties to family and community are a domain of the CDoH, where society is constructed around community, kinship, and family and being part of the community may necessitate responsibilities and obligations (Bourke et al., 2018; Finlay et al., 2020). Connections were not only to maintain personal contact but involved connections to community—viewed as an extension of the family—a concept also described by Pace and Grenier (2017). They reviewed perceptions of ageing in North American Indigenous Peoples and found that relationships with family and community were fundamental to successful ageing.

Torres Strait Islander identity also aligns with the domain of the CDoH described as “cultural expression and continuity” (Finlay et al., 2020). Participants placed great emphasis on how practicing their cultural activities and traditions supported ageing well. First Nations Peoples have for millennia asserted that practicing culture is fundamental to good health and well-being. This concept has not always been accepted in Western models of health care or research (Finlay et al., 2020). Understanding the role that culture plays is an important aspect in any framework that seeks to understand the ageing experience for First Nations Peoples living in the Torres Strait and NPA. Furthermore, an understanding of the importance of culture as a determinant of health and well-being needs to progress and be recognized as a significant factor in Indigenous health and well-being, if health inequities are to be addressed (Parter et al., 2024).

Living a healthy lifestyle symbolized a holistic approach to ageing well, where mental health, physical activity, and diet were all in a positive balance, and health and ageing flourished. When any one of those factors was negatively impacted, health and the ability to age well were hindered. Findings from this study are consistent with the literature on global Indigenous ageing that found it was a combination of factors across mental, physical, spiritual, and emotional realms that supported a person to age well (Quigley et al., 2022). A holistic approach to ageing well is needed in the design of effective policies, programs, and support for the growing cohort of ageing First Nations Peoples in the Torres Strait and NPA.

Strong leadership was significant to the participants in ageing well. This theme aligns with the domain of “Self-determination and leadership” with the CDoH (Finlay et al., 2020). Leadership roles have been seen to strengthen cultural affiliations, provide a sense of purpose, elicit feelings of being needed and respected, and instill a sense of pride in older Indigenous adults (Athira et al., 2024; Coombes et al., 2018; McCausland et al., 2023; Quigley et al., 2022). Older adults were generally shown respect within their communities, contrary to Western perceptions of ageing where older adults are often perceived as a burden on society (Dionigi, 2015; Quigley et al., 2022). The sociocultural aspects of the roles of leaders in Indigenous communities suggest differing values and priorities to ageing well, to those of mainstream frameworks, and needs to be acknowledged in policy and service delivery (Yashadhana et al., 2021).

Passing on knowledge, tradition, and cultural practices supported ageing well. This aligns with “Indigenous beliefs and knowledge” and “Indigenous language” in the CDoH (Finlay et al., 2020). Passing on traditional values, languages, beliefs, wisdom, skills, and knowledge and how this promotes ageing well has been described in previous literature (Coombs et al., 2018; Pace & Grenier, 2017; Quigley et al., 2022). This study extends the literature highlighting important determinants for ageing well for Indigenous Peoples are ideological and culturally situated rather than based on gaining materialistic wealth, and achieving good health as indicators of ageing well, associated with Western views of successful ageing (Quigley et al., 2022).

These findings evidence elements of CDoH that are significant contributors to ageing well and are protective factors in that trajectory. However, challenges to ageing well were significant for First Nations People living in the Torres Strait. Participants shared how the impacts of colonization are widespread, including ill health, substance abuse, and destruction of traditional lifestyles and practices, which have diluted Torres Strait Peoples’ culture. For some, an internal dilemma arises over how to integrate modern technologies, like smartphones, in a way that aligns with traditional practices. Within Indigenous communities, there is a divide between those who view technology and the Internet as opportunities and those who perceive them as threats to the existence and dignity of Indigenous Peoples. These perspectives are influenced by the lasting effects of colonialism and the continuous efforts of Indigenous communities to protect their cultural heritage and dignity (Sianturi et al., 2023). Impacts of colonization on the health and well-being of First Nations Peoples in Australia are well documented (Dudgeon et al., 2010; Paradies, 2016; Sherwood, 2013). Findings from this study are consistent with literature exploring ageing within Aboriginal communities in Australia, and how the ongoing legacy of colonization influences the ability to age well (Coombes et al., 2018; McCausland et al., 2023; Radford et al., 2019; Yashadhana et al., 2021). These impacts of colonization intersect with the broader SDoH that exacerbate ongoing inequity. In this study, these included housing issues, environmental challenges, cost of living, access to transport, food security, and access to culturally appropriate health and aged care services—a theme consistent with Indigenous Peoples internationally (Quigley et al., 2022). However, participants’ inner strength, evidenced through resilience, attitudes, personal attributes, and outlook on ageing, counterbalanced difficulties faced, meaning that ageing well, for many, is achievable. These findings resonate within the literature on Indigenous ageing globally (Pace & Grenier, 2017; Quigley et al., 2022; Yashadhana et al., 2021).

### Implications of Findings

Voicing First Nation People’s perceptions of and priorities for ageing well is an essential element for the delivery of person-centered care to address health inequities (Coombes et al., 2018). Asking community “What does ageing well mean to you?” has provided insights into the importance of incorporating the CDoH into the design of policies, programs, and supports to improve the ability to age well for residents of the Torres Strait region of Australia.

Embedding CDoH into health policy and practice will require systemic change and Indigenous leadership (Finlay et al., 2020). At present, the disconnect between Indigenous culture and Western health care models adversely affects ageing well for many First Nations People (Coombes et al., 2018). Ageing well programs and supports must take a culturally safe, holistic, multifaceted, and whole-of-community approach (McCausland et al., 2023; Quigley et al., 2022; Wettasinghe et al., 2020) and address inequities across the life course, including the wider SDoH, that influence ageing (Pace & Grenier, 2017; Quigley et al., 2022; WHO, 2015). Harnessing the strengths of individuals, their resilience, attitudes, and approaches to life, with those of community, promotes a strengths-based approach to ageing well (Quigley et al., 2022).

### Limitations

Yarning circles were held in three of the five island clusters across the Torres Strait. Given the diversity across the region, findings may not represent perspectives from other Torres Strait communities, although many participants spoke about family connections to island homes not included in this study. There may have been other opinions that the researchers did not capture, as those who attended were those who were interested in talking about their health and ageing and were proactive in wanting to make changes. Participants involved were also physically well enough to leave their homes to attend the yarns.

## Conclusion

These findings broaden the current understanding of ageing well in a wider field of Indigenous ageing to be inclusive of Torres Strait Islander Peoples’ knowledge and perceptions. The concept of ageing well is deeply rooted in the CDoH, with emphasis on connections to island home, family, and community. Specifically, factors that kept people strong and ultimately led to them being able to age well included: maintaining a strong Torres Strait identity and Torres Strait way of life through practicing of culture, and traditions, including the passing on of knowledge and wisdom; balancing physical, mental, cultural, and spiritual domains; and having strong community leadership. By centering First Nations perspectives in policies and practices aimed at promoting health in later life, we can improve conditions for enhancing the quality of life for older adults.

## References

[bibr1-10497323251335210] Athira V. H. Nalini R. Krishna Kumar K. (2024). Harnessing resilience in the healthy ageing discourse: Insights from Attappadi Indigenous older adults, Kerala, India. Journal of Gerontological Social Work, 67(8), 1127–1152. 10.1080/01634372.2024.2351074 38739408

[bibr2-10497323251335210] Atkinson P. Baird M. Adams K. (2021). Are you really using yarning research? Mapping social and family yarning to strengthen yarning research quality. AlterNative: An International Journal of Indigenous Peoples, 17(2), 191–201. 10.1177/11771801211015442

[bibr3-10497323251335210] Barlo S. Boyd W. Pelizzon A. Wilson S. (2020). Yarning as protected space: Principles and protocols. AlterNative: An International Journal of Indigenous Peoples, 16(2), 90–98. 10.1177/1177180120917480

[bibr4-10497323251335210] Bessarab D. Ng’andu B. (2010). Yarning about yarning as a legitimate method in Indigenous research. International Journal of Critical Indigenous Studies, 3(1), 37–50. 10.5204/ijcis.v3i1.57

[bibr5-10497323251335210] Bourke S. Wright A. Guthrie J. Russell L. Dunbar T. Lovett R. (2018). Evidence review of Indigenous culture for health and wellbeing. The International Journal of Health, Wellness, and Society, 8(4), 11–27. 10.18848/2156-8960/CGP/v08i04/11-27

[bibr6-10497323251335210] Braun V. Clarke V. (2022). Thematic analysis: A practical guide. Sage Publications.

[bibr8-10497323251335210] Browne C. V. Braun K. L. (2017). Away from the islands: Diaspora’s effects on native Hawaiian elders and families in California. Journal of Cross-Cultural Gerontology, 32(4), 395–411. 10.1007/s10823-017-9335-3 29032489

[bibr9-10497323251335210] Bryant J. Bolt R. Botfield J. Martin K. Doyle M. Murphy D. Graham S. Newman C. Bell S. Treloar C. Browne A. Aggleton P. (2021). Beyond deficit: ‘Strengths-based approaches’ in Indigenous health research. Sociology of Health & Illness, 43(6), 1405–1421. 10.1111/1467-9566.13311 34145599

[bibr10-10497323251335210] Butcher E. Breheny M. (2016). Dependence on place: A source of autonomy in later life for older Māori. Journal of Aging Studies, 37, 48–58. 10.1016/j.jaging.2016.02.004 27131278

[bibr11-10497323251335210] Byrne A. McLellan S. Willis E. Curnow V. Harvey C. Brown J. Hegney D. (2021). Yarning as an interview method for non-Indigenous clinicians and health researchers. Qualitative Health Research, 31(7), 1345–1357. 10.1177/1049732321995802 33645333

[bibr12-10497323251335210] Commonwealth of Australia . (2018). National Health and Medical Research Council, Ethical conduct in research with Aboriginal and Torres Strait Islander peoples and communities: Guidelines for researchers and stakeholders. Commonwealth of Australia.

[bibr13-10497323251335210] Coombes J. Lukaszyk C. Sherrington C. Keay L. Tiedemann A. Moore R. Ivers R. (2018). First Nation Elders’ perspectives on healthy ageing in NSW, Australia. Australian & New Zealand Journal of Public Health, 42(4), 361–364. 10.1111/1753-6405.12796 29888836

[bibr14-10497323251335210] Department of Health . (2021). National Aboriginal and Torres Strait Islander health plan 2021-2031. Publications Number DT0002195. The Commonwealth of Australia.

[bibr15-10497323251335210] Dionigi R. (2015). Stereotypes of aging: Their effects on the health of older adults. Journal of Geriatrics, 2015, 954027. 10.1155/2015/954027

[bibr16-10497323251335210] Dudgeon P. Wright M. Paradies Y. Garvey D. Walker I. (2010). Aboriginal social, cultural and historical contexts. In Dudgeon P. Milroy H. Walker R. (Eds.), Working together: Aboriginal and Torres Strait Islander mental health and wellbeing principles and practice. Department of Health and Ageing.

[bibr17-10497323251335210] Finlay S. M. Canuto K. Canuto K. Neal N. Lovett R. (2020). Aboriginal and Torres Strait Islander connection to culture: Building stronger individual and collective wellbeing. Medical Journal of Australia, 214(8 Suppl), S12–S16. https://doi.org/10.5694mja2.51020

[bibr18-10497323251335210] Fogarty W. Lovell M. Langenberg J. Heron M.-J. (2018). Deficit discourse and strengths-based approaches: Changing the narrative of Aboriginal and Torres Strait Islander health and wellbeing. The Lowitja Institute.

[bibr19-10497323251335210] Geia L. Hayes B. Usher K. (2013). Yarning/Aboriginal storytelling: Towards an understanding of an Indigenous perspective and its implications for research practice. Contemporary Nurse, 46(1), 13–17. 10.5172/conu.2013.46.1.13 24716757

[bibr20-10497323251335210] Kingsley J. Townsend M. Henderson-Wilson C. Bolam B. (2013). Developing an exploratory framework linking Australian Aboriginal Peoples’ connection to country and concepts of wellbeing. International Journal of Environmental Research and Public Health, 10(2), 678–698. 10.3390/ijerph10020678 23435590 PMC3635170

[bibr21-10497323251335210] Lawrence D. Lawrence H. R. (2004). Torres Strait: The region and its people. In Davis R. (Ed.), Woven histories, dancing lives: Torres Strait Islander identity, culture and history (pp. 15–29). Aboriginal Studies Press. 10.3316/informit.085575432X

[bibr22-10497323251335210] Mafileo T. Vaka S. Leau K. Satele P. Alefaio S. (2024). Decolonising qualitative analysis: Collectively weaving understanding using Talanoa and Fa’afaletui Pacific-Indigenous research methods. International Journal of Qualitative Methods, 23(1), 1–10. 10.1177/16094069241272241

[bibr23-10497323251335210] Mam S. Elu M. Trevallion I. Reid A. (1993). The coconut palm tree-metaphor for Islander family life. Family Matters, 35, 19–21. https://aifs.gov.au/research/family-matters/no-35/torres-strait-islander-family-life

[bibr24-10497323251335210] McCausland R. Jamieson S. Robinson V. Spencer W. MacGillivray P. Andersen M. (2023). Elders’ perspectives and priorities for ageing well in a remote Aboriginal community. Ageing and Society, 45(1), 31–54. 10.1017/S0144686X23000156

[bibr25-10497323251335210] Murrup-Stewart C. Whyman T. Jobson L. Adams K. (2021). “Connection to culture is like a massive lifeline”: Yarning with Aboriginal young people about culture and social and emotional wellbeing. Qualitative Health Research, 31(10), 1833–1846. 10.1177/10497323211009475 33938295

[bibr26-10497323251335210] Pace J. (2020). “Place-ing” dementia prevention and care in NunatuKavut, Labrador. Canadian Journal on Aging, 39(2), 247–262. 10.1017/S0714980819000576 31666149

[bibr27-10497323251335210] Pace J. Grenier A. (2017). Expanding the circle of knowledge: Reconceptualizing successful aging among North American older Indigenous Peoples. The Journals of Gerontology, 72(2), 248–258. 10.1093/geronb/gbw128 27729385

[bibr28-10497323251335210] Paradies Y. (2016). Colonisation, racism and indigenous health. Journal of Population Research, 33(1), 83–96. 10.1007/s12546-016-9159-y

[bibr29-10497323251335210] Parter C. Murray D. Mokak R. Briscoe K. Weston R. Mohamed J. (2024). Implementing the cultural determinants of health: Our knowledges and cultures in a health system that is not free of racism. Medical Journal of Australia, 221(1), 5–7. 10.5694/mja2.52352 38946631

[bibr30-10497323251335210] Quigley R. Russell S. G. Larkins S. Taylor S. Sagigi B. Strivens E. Redman-MacLaren M. (2022). Aging well for Indigenous Peoples: A scoping review. Frontiers in Public Health, 10, Article 780898. 10.3389/fpubh.2022.780898 35223727 PMC8866315

[bibr31-10497323251335210] Quigley R. Russell S. G. Sagigi B. Miller G. Strivens E. (2021). Community involvement to maximise research success in Torres Strait Islander populations: More than just ticking the boxes. Rural and Remote Health, 21(3), Article 5957. 10.22605/RRH5957 34275323

[bibr32-10497323251335210] Radford K. Allan W. Donovan T. Delbaere K. Garvey G. Broe G. Daylight G. Anderson M. Timbery A. Sullivan K. Nichols M. Lavrencic L. (2019). Sharing the wisdom of our elders final report. Neuroscience Research Australia.

[bibr33-10497323251335210] Sadana R. Blas E. Budhwani S. Koller T. Paraje G. (2016). Healthy ageing: Raising awareness of inequalities, determinants, and what could be done to improve health equity. The Gerontologist, 56(Suppl 2), S178–S193. 10.1093/geront/gnw034 26994259

[bibr34-10497323251335210] Shay M. (2019). Extending the yarning yarn: Collaborative Yarning Methodology for ethical Indigenist education research. The Australian Journal of Indigenous Education, 50(1), 62–70. 10.1017/jie.2018.25

[bibr35-10497323251335210] Sherwood J. (2013). Colonisation - It’s bad for your health: The context of Aboriginal health. Contemporary Nurse, 46(1), 28–40. 10.5172/conu.2013.46.1.28 24716759

[bibr36-10497323251335210] Sianturi M. Lee J.-S. Cumming T. M. (2023). Using technology to facilitate partnerships between schools and Indigenous parents: A narrative review. Education and Information Technologies, 28(5), 6141–6164. 10.1007/s10639-022-11427-4

[bibr37-10497323251335210] Smith R. Devine S. Preston R. (2020). Recommended methodologies to determine Australian Indigenous community members’ perceptions of their health needs: A literature review. Australian Journal of Primary Health, 26(2), 95–103. 10.1071/PY19078 32061267

[bibr38-10497323251335210] Walker M. Fredericks B. Mills K. Anderson D. (2013). “Yarning” as a method for community-based health research with Indigenous women: The Indigenous Women’s Wellness Research Program. Health Care for Women International, 35(10), 1216–1226. 10.1080/07399332.2013.815754 23980668

[bibr39-10497323251335210] Wettasinghe P. Allan W. Garvey G. Timbery A. Hoskins S. Veinovic M. Daylight G. Mack H. Minogue C. Donovan T. Broe G. Radford K. Delbaere K. (2020). Older Aboriginal Australians’ health concerns and preferences for healthy ageing programs. International Journal of Environmental Research and Public Health, 17(20), Article 7390. 10.3390/ijerph17207390 33050541 PMC7600369

[bibr40-10497323251335210] World Health Organization . (2015). World report on ageing and health.

[bibr41-10497323251335210] World Health Organization . (2018). Ageing and health. https://www.who.int/news-room/fact-sheets/detail/ageing-and-health

[bibr42-10497323251335210] Yashadhana A. Howie A. Veber M. Cullen P. Withall A. Lewis E. McCausland R. Macniven R. Andersen M. (2021). Experiences and perceptions of ageing among older First Nations Australians: A rapid review. Australasian Journal on Ageing, 41(1), 8–19. 10.1111/ajag.13031 34905642

